# Time-Lapse Imaging of Red Blood Cell Invasion by the Rodent Malaria Parasite *Plasmodium yoelii*

**DOI:** 10.1371/journal.pone.0050780

**Published:** 2012-12-05

**Authors:** Kazuhide Yahata, Moritz Treeck, Richard Culleton, Tim-Wolf Gilberger, Osamu Kaneko

**Affiliations:** 1 Department of Protozoology, Institute of Tropical Medicine (NEKKEN) and the Global COE Program, Nagasaki University, Sakamoto, Nagasaki, Japan; 2 Department of Molecular Parasitology, Bernhard Nocht Institute for Tropical Medicine, Hamburg, Germany; 3 Department of Microbiology and Immunology, Stanford University School of Medicine, Stanford, California, United States of America; 4 Malaria Unit, Institute of Tropical Medicine (NEKKEN), Nagasaki University, Nagasaki, Japan; 5 Department of Pathology and Molecular Medicine, M.G. DeGroote Institute for Infectious Disease Research, McMaster University, Hamilton, Ontario, Canada; University of Bern, Switzerland

## Abstract

In order to propagate within the mammalian host, malaria parasites must invade red blood cells (RBCs). This process offers a window of opportunity in which to target the parasite with drugs or vaccines. However, most of the studies relating to RBC invasion have analyzed the molecular interactions of parasite proteins with host cells under static conditions, and the dynamics of these interactions remain largely unstudied. Time-lapse imaging of RBC invasion is a powerful technique to investigate cell invasion and has been reported for *Plasmodium knowlesi* and *Plasmodium falciparum.* However, experimental modification of genetic loci is laborious and time consuming for these species. We have established a system of time-lapse imaging for the rodent malaria parasite *Plasmodium yoelii*, for which modification of genetic loci is quicker and simpler. We compared the kinetics of RBC invasion by *P. yoelii* with that of *P. falciparum* and found that the overall kinetics during invasion were similar, with some exceptions. The most striking of these differences is that, following egress from the RBC, the shape of *P. yoelii* merozoites gradually changes from flat elongated ovals to spherical bodies, a process taking about 60 sec. During this period merozoites were able to attach to and deform the RBC membrane, but were not able to reorient and invade. We propose that this morphological change of *P. yoelii* merozoites may be related to the secretion or activation of invasion-related proteins. Thus the *P. yoelii* merozoite appears to be an excellent model to analyze the molecular dynamics of RBC invasion, particularly during the morphological transition phase, which could serve as an expanded window that cannot be observed in *P. falciparum*.

## Introduction

The clinical symptoms of malaria manifest during the erthrocytic cycle of *Plasmodium* infection, during which the parasites invade red blood cells (RBCs), and replicate within them. RBC invasion is a rapid and complex process governed by specific molecular interactions between parasite derived molecules and RBC components [1], [2]. This invasion process has previously been divided into three distinct phases based on observations of time-lapse images of *Plasmodium knowlesi* and *Plasmodium falciparum* [3], [4]. Firstly, the merozoite binds to the RBC surface and reorients, bringing the anterior end into contact with the RBC membrane (Pre-invasion phase). Secondly, a tight junction is formed between the parasite’s anterior end and the RBC membrane, and the merozoite moves into the RBC (Invasion phase). Finally, a parasitophorous vacuole is established around the parasite and dynamic morphological deformation of the RBC occurs (Echinocytosis phase). Many parasite molecules involved in this process have been identified, yet the dynamic relationship between these molecules and RBC components during invasion are not well understood [5].

Time-lapse imaging of RBC invasion is a powerful technique for studying host-parasite interactions during cell invasion. The first imaging of RBC invasion by a malaria parasite was reported by Dvorak et al in 1975, using the primate malaria parasite *P. knowlesi*. Imaging of *P. falciparum* was not reported until 2009 [4], [6]. Additionally, it has recently become possible to observe the effects of invasion inhibitory reagents, such as chemical compounds and antibodies against vaccine candidate proteins, using wild type or genetically modified *P. falciparum* parasite lines [6], [7]. However, genetic modification of *P. falciparum* is laborious and time consuming. Thus, in order to study the basic biology of malaria parasites, such as the kinetics of parasite molecules during invasion, rodent malaria parasites may have advantages, as stable transformation and gene targeting are quick and well established for *Plasmodium berghei* and *Plasmodium yoelii* [8], [9]. In addition, *P. yoelii* has a variety of lines with distinct RBC preference and virulence characteristics [10], the whole life-cycle including mosquito-stages can be maintained in the laboratory with relative ease, genetic crosses of *P. yoelii* are easily produced [11], [12], many antibodies against invasion-related molecules have been produced, and the genome sequence is available for the 17XNL clone1.1 (17X1.1) line [13]. Although continuous culture of rodent malaria parasites has not yet been achieved, short-term culture from ring to schizont stage is possible for *P. berghei*, *P. yoelii* and *P. chabaudi* [14], [15], [16]. Here we report the establishment of time-lapse imaging for *P. yoelii* in order to observe RBC invasion in real-time using two distinct *P. yoelii* strains. We observed the parasites from rupture to invasion of RBCs for the rapid growth rate lethal line *P. yoelii* 17XL and the non-lethal line *P. yoelii* 17X1.1 that shows intermediate growth phenotype between 17XL and slow growing non-lethal line 17XNL, and compared the kinetics of RBC invasion with that of *P. falciparum.*

## Results

### Rupture of the *P. yoelii*-infected RBC (iRBC) and Merozoite Release

**Figure 1:**
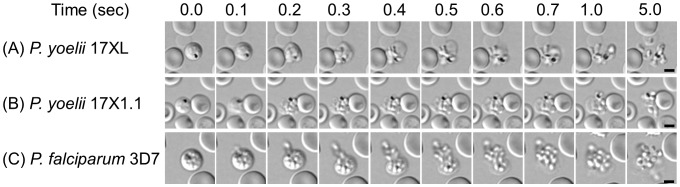
Time-lapse imaging for the rupture of schizont-infected red blood cells. Images were captured every 0.1 sec with transmitted light for *Plasmodium yoelii* 17XL (A), *P. yoelii* 17X1.1 (B), and *Plasmodium falciparum* 3D7 line (C). The bars represent 5 µm.

Time-lapse imaging with transmitted light of segmented-schizont of *P. yoelii* and *P. falciparum* revealed that the time from the rupture of the schizont-iRBC to merozoite release occurred within 1 second (Fig. 1 and Movies S1, S2 and S3). The diameters of the segmented-schizont-iRBC of *P. yoelii* 17XL and 17X1.1 were 5.4±0.2 (mean ± SD) and 5.3±0.3 µm, respectively, slightly smaller than that of *P. falciparum* (5.8±0.2 µm). The individual merozoites in the segmented-schizont stage of *P. falciparum* were visible under the light microscope and always surrounded the haemozoin located in the center of parasite-iRBC (Fig. 1C, 0.0 sec), and intracellular merozoites were concentrated towards the haemozoin immediately prior to RBC rupture (Fig. 1C, 0.1 sec and Movies S3). In contrast, intracellular merozoites in the segmented-schizont stage of both *P. yoelii* lines were not visible by transmitted light until rupture, thus their motion before RBC rupture was unable to be assessed (Fig. 1A and 1B, 0.0 sec and Movie S1 and S2). The median numbers of *P. yoelii* 17XL and *P. yoelii* 17X1.1 merozoites were 8 (n = 20 mono-infected iRBCs), less than *P. falciparum* merozoites was 20 (16–28; n = 20). When *P. falciparum* and *P. yoelii*-iRBC ruptured, breakage occurred at a single point on the surface of the RBC from which merozoites were released. The broken RBC membrane was observed clinging to the released merozoites (Fig. 1A, 1B and 1C, 0.2 to 1.0 sec). In conclusion, the progress of 2 lines of *P. yoelii*-iRBC rupture was similar with *P. falciparum*.

### Three-phase Process and Kinetic Difference in RBC Invasion between *P. yoelii* and *P. falciparum*

**Figure 2:**
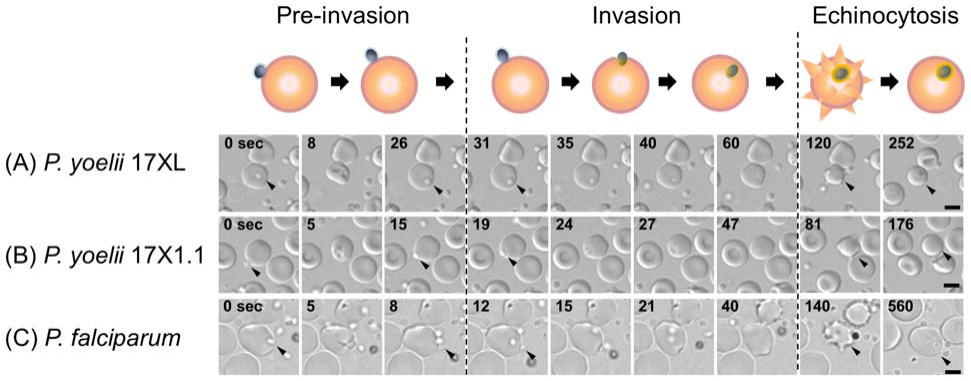
Three phase processes of the red blood cell (RBC) invasion by *Plasmodium yoelii*. Time-lapse imaging of RBC invasion was captured every 0.1 sec with transmitted light for *P. yoelii* 17XL (A), *P. yoelii* 17X1.1 (B), and *Plasmodium falciparum* 3D7 line (C). First “Pre-invasion” phase started from the initial attachment between the merozoite (0 second, arrow head) and RBC plasma membrane, followed by the RBC deformation, and apical reorientation of the merozoite (rightmost column of “Pre-invasion” phase). Second “Invasion” phase consisted of the internalization of a merozoite into RBC and a rapid rotary movement of the internalized merozoite (arrow). Final “Echinocytosis” phase was defined as RBC being deformed to spike-like shape. The bars represent 5 µm.

**Figure 3:**
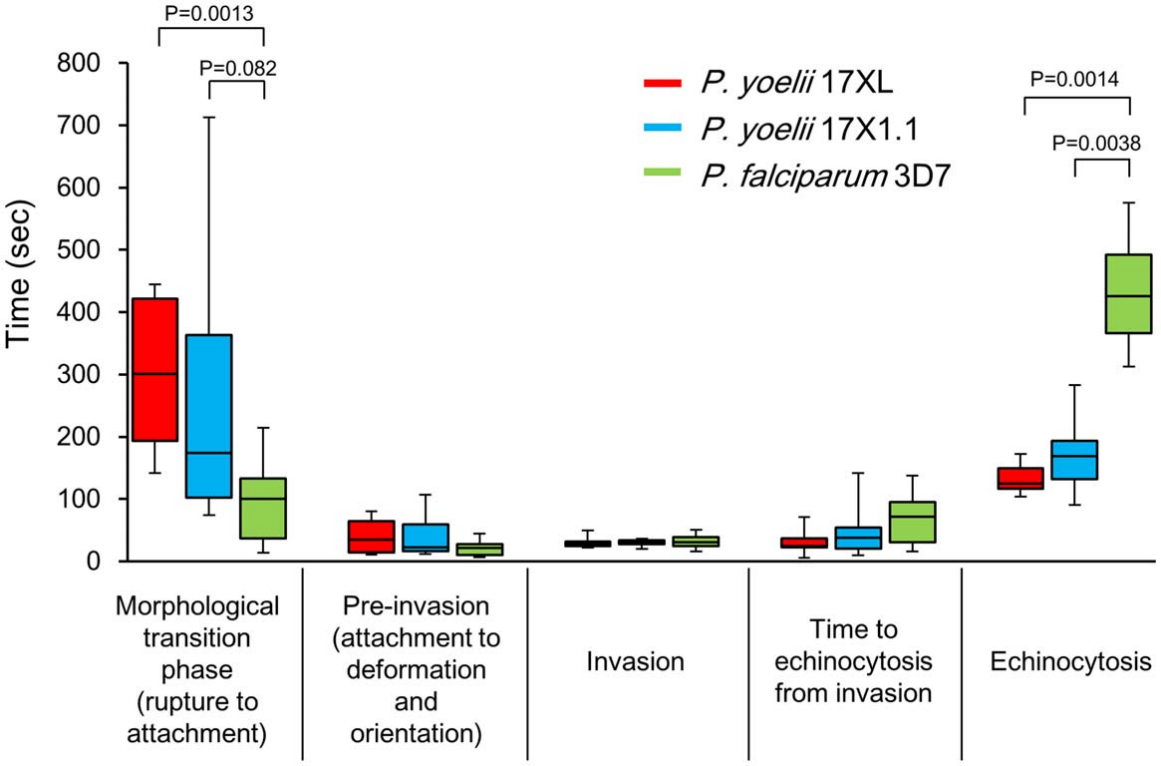
Kinetic difference in red blood cell (RBC) invasion between *Plasmodium* species. The median time for each step are shown as a box plot with whiskers from minimum to maximum. The interquartile range shows as box with the median marked as a horizontal line, minimum and maximum from lower and upper quartile represent error bar. *P* values were determined using the Mann-Whitney U test. See Table S1 for detail values.

RBC invasion of *P. yoelii* is morphologically similar to *P. falciparum* and *P. knowlesi*, and the previously proposed three phase processes (Pre-invasion, Invasion, and Echinocytosis phases; [4]) can also be applied (Fig. 2). To compare the kinetics of the *P. yoelii* RBC invasion with those of *P. falciparum*, we determined the time of each invasion step for *P. yoelii* and *P. falciparum*. Using 9–12 invasion events, the median time for each step was obtained (Fig. 3, Table S1). The median time from RBC rupture to the initial attachment of *P. yoelii* merozoites were 300 seconds (range: 142–445, 17XL) and 173 sec (74–713, 17X1.1), which were longer than that of *P. falciparum* 3D7 line (Fig. 3, 100 sec, 14–214). *P. yoelii* merozoites remained viable for longer than those of the *P. falciparum* 3D7 line following egress, demonstrated by the observation that *P. yoelii* merozoites were able to invade RBCs up to 445 sec (17XL) and 713 sec (17X1.1) after release, whereas merozoites of *P. falciparum* were not able to invade following 214 sec after release (Table S1). After the initial attachment of *P. yoelii* and *P. falciparum*, RBC deformation started immediately (Fig. 2, 0 sec), followed by an apical reorientation of the merozoite (“resting phase”) for around 10 sec at the contact site (Fig. 2, 26 sec for *P. yoelii* 17XL, 15 sec for *P. yoelii* 17X1.1, and 8 sec for *P. falciparum*). The time of the “pre-invasion” phase, consisting of attachment to and deformation of the RBC and merozoite reorientation, for *P. yoelii* was 35 sec (11–80, 17XL) and 22 sec (12–107, 17X1.1), whereas that for *P. falciparum* was 21 sec (7–44). Following the resting phase, merozoites invade RBCs from their apical end, and this “invasion” phase of *P. yoelii* took 29 sec (22–50, 17XL) and 30 sec (20–36, 17X1.1) similar to *P. falciparum* (Fig. 3, 30 sec, 16–51). Following internalization, and prior to rapid rotation of the merozoite within the iRBC, the RBC deforms to form a spike-like structure; a process known as the “Echinocytosis” phase (Fig. 2, 120 to 252 sec for *P. yoelii* 17XL, 81 to 176 sec for *P. yoelii* 17X1.1, and 140 to 560 sec for *P. falciparum*). This phase began 30 to 70 sec after internalization. The duration of echinocytosis in *P. yoelii* was 125 sec (104–172, 17XL) and 168 sec (91–283, 17X1.1), whereas that of *P. falciparum* was significantly longer (426 sec, 313–576, Fig. 3). Merozoites undergo rapid rotation within the iRBC during echinocytosis which ceases towards the end of the phase (Movie S1, S2 and S3). The shape of the *P. falciparum*-iRBC normalized following completion of the invasion process, and the parasite quickly transformed into an amoeboid ring-stage; a process which had already started during the echinocytosis phase (Movie S3). In contrast, the majority of the RBCs targeted for invasion by *P. yoelii* merozoites lysed following the echinocytosis phase and the parasite could not grow further, which could be one reason why continuous robust *in vitro* culture can not be achieved for *P. yoelii* (Movie S4), reflecting some initial observations for *P. knowlesi* invasion [3]. The apical end of the *P. yoelii* merozoite was observed to be firmly attached to the lysed RBC membrane, suggesting that a tight junction was irreversibly formed.

### Morphological Transition of *P. yoelii* Merozoite after Released from RBC

**Figure 4:**
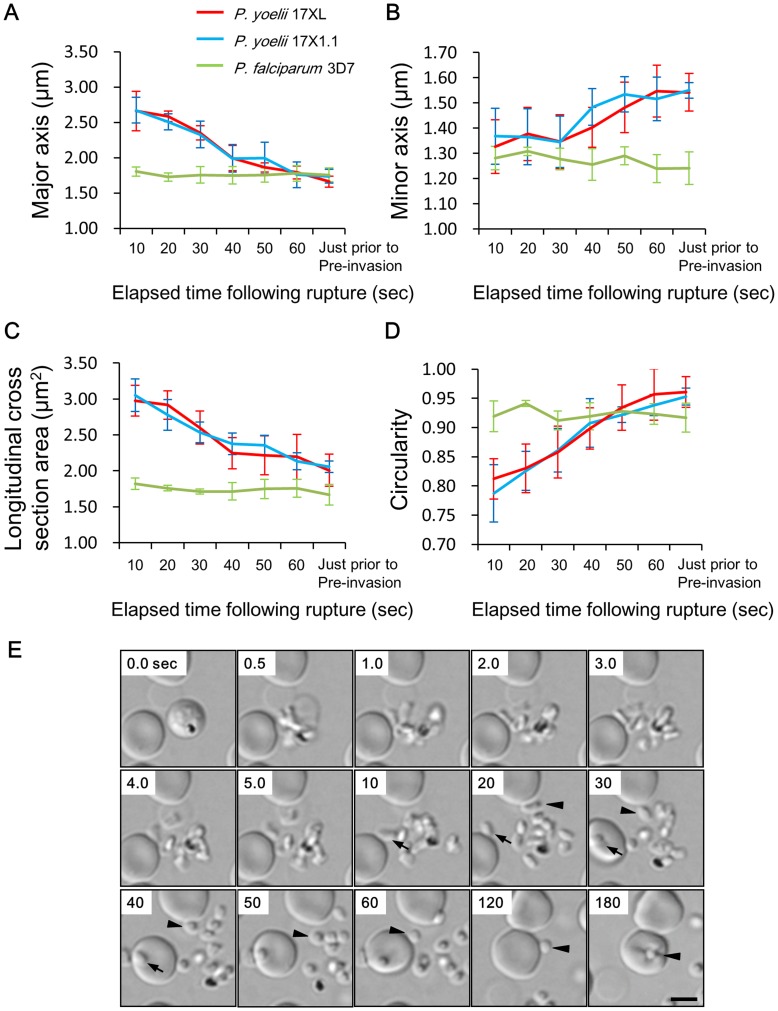
Morphological change of the *Plasmodium yoelii* merozoite after released from red blood cell (RBC). The major axis (A), minor axis (B), longitudinal cross section area (C), and circularity (D) were measured every 10 sec from RBC rupture to pre-invasion for invasive merozoites (n = 9–12). The average and the error representing one standard deviation were plotted in the line charts. Circularity was calculated using the following formula: Circularity = 4πArea/Perimeter^2^. A value of 1 indicates a perfect circle and the value of 0 indicates an increasingly elongated polygon. See Table S1 for detail values. (E) Time-lapse sequence of merozoite release of *P. yoelii* 17XL was recorded every 0.1 sec. Arrowhead indicates same invasive merozoite in the sequence and the arrow indicates an attachment of an immature flat elongated oval merozoite. A mature spherical invasive merozoite attached to the RBC and deformed RBC (Pre-invasion) at 180 sec. The bar represents 5 µm.

After release from the RBC, the shape of *P. yoelii* merozoites gradually changed from a flat elongated oval (Fig. 4E, 20 sec) to spherical (Fig. 4E, 60 sec), which took about 60 sec. We named this phase the “morphological transition phase”, whereas the shape of the *P. falciparum* merozoite was already spherical when it was released and did not change through time (Fig. 1 and Movie S1, S2 and S3). The major axis, minor axis and the longitudinal cross section area of *P. yoelii* 17XL invasive merozoites gradually reduced from 2.67±0.18 (mean ± SD) µm to 1.66±0.10 µm, 1.33±0.11 µm to 1.54±0.07 µm and 2.98±0.22 µm^2^ to 2.01±0.23 µm^2^, respectively (Fig. 4A, 4B and 4C). In contrast, those of *P. falciparum* invasive merozoites did not change dramatically (1.81±0.06 µm to 1.76±0.10 µm, 1.28±0.05 µm to 1.24±0.06 µm and 1.82±0.08 µm^2^ to 1.67±0.14 µm^2^, respectively). The circularity of *P. yoelii* invasive merozoites gradually increased from 0.79±0.03 µm to 0.95±0.03 µm (17XL) and 0.81±0.05 µm to 0.96±0.01 µm (17X1.1), indicating that the shape of *P. yoelii* invasive merozoite became more spherical, whereas that of *P. falciparum* invasive merozoites was consistent, starting with 0.92±0.03 µm and ending with 0.92±0.02 µm. The non-invasive merozoites for which RBC invasion was not observed within 20 minutes of RBC rupture displayed a similar morphological transition phase (Table S2). The difference of the merozoite shape between late schizont of intraerythrocytic and free merozoites released from ruptured schizonts of *P. yoelii nigeriensis* has been shown by electron microscopy [17], and the movie of *P. knowlesi* also shows morphological transition after release [3], which is consistent with our observations. Morphological changes of *P. yoelii* merozoites appeared to be related to the time from the merozoite release to the initial attachment for the successful RBC invasion, for which the fastest time was 142 sec for 17XL (Table S1, median 300 sec and longest 445 sec) and 74 sec for 17X1.1 (median 173 sec and longest 713 sec), whereas only 14 sec was required for *P. falciparum* (median 100 sec and longest 214 sec). Although the flat elongated oval-shaped merozoites were able to attach to RBC within a minute after release, most of them were unable to deform the RBC, and detached from the cell (Fig. 4E, 10 of 12 merozoites for *P. yoelii* 17XL and 9 of 9 merozoites for *P. yoelii* 17X1.1). Some merozoites were able to deform the RBC, but did not re-orientate and also detached from the RBC surface (Fig. 4E arrows and Movie S1 left bottom, 2 of 12 merozoites for *P. yoelii* 17XL attached and deformed RBC within 60 sec after release), whereas spherical merozoites were able to deform RBC and invade after attachment.

## Discussion

We established a stable time-lapse imaging system for RBC invasion by the rodent malaria parasite *P. yoelii*. Using this system, we were able to observe, for the first time, the rupture of *P. yoelii*-iRBC. That this process has not been observed for rodent malaria parasites previously may be due to the fact that *P. berghei,* another commonly used rodent malaria parasite, generally arrests at the late schizont stage and *P. berghei*-iRBCs have not been observed to rupture in vitro [14]. Spontaneous rupture was observed for mature RBCs infected with *P. yoelii* 17XL, YM, and 17X1.1 lines, all of which invade and grow in mature RBCs as well as reticulocytes. However, no rupture was observed of reticulocytes infected with the reticulocyte-restricted lines *P. yoelii* 17XNL and *P. yoelii* CU during numerous experiments. RBC membrane compositions change during erythroid differentiation [18], and it is possible that mature mouse RBC membranes may be more easily ruptured by schizont stage rodent malaria parasites than reticulocyte membranes. Physiological shear stresses present in vivo may be required to release merozoites from reticulocytes during infection. We also observed that *P. yoelii*-iRBCs containing freely moving merozoites never ruptured. It is possible that the parasitophorous vacuole or the RBC membrane may be damaged due to decreased density of the iRBC cytosol, a phenomenon previously observed for *P. falciparum* [19]. Rupture of iRBCs relies on parasite proteases, including the cytoskeleton-degrading malarial proteases falcipain-2, plasmepsin II and papain-like protease, serine-rich antigen (SERA) in the parasitophorous vacuole [20]. The activation of the SERA proteases is regulated by a subtilisin-like serine protease, SUB1 [21], resulting a sequential breakdown of the parasitophorous vacuole and RBC membrane. Thus the inadvertent rupture of the parasitophorus vacuole membrane or RBC membrane may result in inactivation of the enzymes responsible for successful rupture.

We found that the kinetics of RBC invasion was similar between the rapid growth rate *P. yoelii* 17XL line and the non-lethal 17X1.1 line (with an intermediate growth phenotype between rapid and slow growing lines), indicating that the kinetics of the invasion were not responsible for the differences in the growth rates of these parasites. The overall kinetics of the RBC invasion by *P. yoelii* was similar to *P. falciparum*, except that 1) *P. yoelii* has a morphological transition phase that occurs prior to the initiation of the previously described three phases of RBC invasion, and 2) the echinocytosis phase of *P. yoelii* is shorter than that of *P. falciparum*. Following egress from the iRBC, *P. falciparum* was able to start to invade RBCs earlier than *P. yoelii*. We found that this difference in time between *P. yoelii* and *P. falciparum* is due to the morphological transition phase observed for *P. yoelii*, during which the merozoite’s shape gradually changes from a flat elongated oval shape to a fully invasive spherical shape. What drives this transition is unclear, but plausible explanations are a rearrangement of cytoskeletal elements or a change in the osmotic properties of the merozoite, in which an increased water content leads to a swelling of the merozoite. Whatever the driving force of this transition, it appears to be critical for the successful invasion by *P. yoelii* merozoites.

We speculate that during the transition phase *P. yoelii* merozoites prepare for invasion by secretion of invasion related ligands. Exposure of *P. falciparum* merozoites to low potassium ion concentrations triggers an increase of intracellular calcium in *P. falciparum* which in turn stimulates secretion of the ligands from micronemes to the merozoite surface and prepares parasites for invasion [22]. For example, apical membrane antigen 1 (AMA1) is secreted on the merozoite surface after release in both *P. yoelii* and *P. falciparum* [23], [24]. The role of AMA1 appears to occur after reorientation, possibly through the formation of a complex with rhoptry neck-derived proteins that are secreted into the RBC after reorientation, as observed in *P. falciparum* [6], [25], [26], [27]. Other proteins involved in invasion are the Erythrocyte-Binding-Like (EBL) and Reticulocyte Binding-Like (RBL) family proteins, for example, *Py*EBL and Py235 in *P. yoelii* and EBA-175 and PfRH4 for *P. falciparum*, both of which appear to locate to the micronemes and do not translocate on the surface of merozoite [28], [29], [30].

It is possible that proteolytic processing of surface proteins could take place during the transition time. In *P. falciparum* the merozoite surface protein 1 (MSP1), the most abundant protein of the merozoite surface and a prime malaria vaccine candidate, needs extensive proteolytic modification for successful invasion [31]. The morphological transition phase of *P. yoelii* may be useful to analyze the timing of the secretion and activation of the invasion-related proteins, which may be difficult to analyze in *P. falciparum*.

Time-lapse imaging of RBC invasion by malaria merozoites is a useful and powerful technique to evaluate the effect of known or potential invasion inhibitory compounds and antibodies, and to analyze the kinetics of parasite molecules by tagging with fluorescent proteins. The unique morphological transition phase and the relative ease with which transgenic rodent malaria parasites can be generated makes them excellent models for studying the kinetics and function of the proteins involved in RBC invasion.

## Materials and Methods

### Parasite Lines and Culture

*P. falciparum* 3D7 was originally obtained from David Walliker at Edinburgh University [32] and maintained with O+ RBCs in RPMI1640 medium (Invitrogen) supplemented with 25 mM HEPES (Sigma), 0.225% sodium bicarbonate (Invitrogen), 0.1 mM hypoxanthine (Sigma), 25 µg/mL gentamicin (Invitrogen), 0.5% AlbuMax I (Invitrogen) basically according to standard procedures [33]. Human RBCs were obtained from the Universitätslinikum Eppendorf, Hamburg, Germany. *P. falciparum* parasites were synchronized to ring stages with 5% sorbitol solution and cultured until they matured into schizont stages [34]. Cultures with a 0.25% RBC concentration in pre-warmed media were used for live imaging. 1×10^6^ lethal line *P. yoelii* 17XL and non-lethal line *P. yoelii* 17X1.1, obtained from Nagasaki University’s BioResource bank (http://www.nbrp.jp/) were intravenously inoculated into CBA mice (SLC Inc., Shizuoka, Japan). Whole blood was collected from the tail at 3 to 5 days post-inoculation when the parasitaemia reached approximately 5%. Five microliters of whole blood were diluted to an RBC concentration of 0.25% with 1 mL of pre-warmed RPMI1640 medium supplemented with 25 mM HEPES, 0.225% sodium bicarbonate, 0.1 mM hypoxanthine, 10 mg/mL gentamicin and 1% AlbuMax I at a pH of 7.4 [9]. Parasite solution of 0.25% RBC concentration was transferred to a 1 µ-Slide I^0.2^ Luer chamber slide (hydrophobic; ibidi, Germany) and used for microscopy within an hour after bleeding. All experiments conducted in this study were approved by the animal care and use committee, Nagasaki University (Permit number: 0912080806-4).

### Video Microscopy

Video microscopy for *P. yoelii* was performed at 37°C using an inverted microscope (Ti-E; Nikon) with 60x oil objective lens (N.A. 1.4). The inverted microscope was configurated to act as a stable time-lapse imaging system with perfect focus system (PFS, Nikon). The water chamber stage (Tokai-Hit) and the objective lens were kept at 37°C with a thermo controller (Tokai-Hit). Cells were observed by differential interference contrast (DIC) at 3V/15W low power of halogen lamp (12V/100W, 7724L, PHILIPS) to minimize the cell damage. Time-lapse images were captured every 0.1 sec using a CCD camera (ORCA-R2; Hamamatsu photonics) and imaged using the NIS-Element Advanced Research imaging software (Nikon). The video microscopy for *P. falciparum* was performed using an inverted microscope (Axiovert 200 M, Carl Zeiss, Germany) and 63x oil objective lens (N.A. 1.4) kept at 37°C with a thermo controller (Carl Zeiss). Images were captured every 0.1 sec using a CCD camera (AxioCam HRm; Carl Zeiss) and were taken by differential interference contrast (DIC) at 3V/15W with a low power halogen lamp (12V/100W, 7724L, PHILIPS) to minimize the cell damage. Movie files were edited with ImageJ software (http://rsb.info.nih.gov/ij/), the number of RBCs and the spacing between them was calculated from the movie at RBC rupture (Table S1). The major axis, minor axis and the area of the released merozoites were measured every 10 sec under the time-lapse microscope, and circularity of the merozoites was calculated using NIS-Elemens Advanced Research imaging software with the following formula: Circularity = 4πArea/Perimeter^2^. A value of 1 indicates a perfect circle and the value of 0 indicates an increasingly elongated polygon.

## Supporting Information

Table S1 **Time kinetics of RBC invasion for different** ***Plasmodium*** **species.** The median, quartile, minimum and maximum time were obtained for each invasion step. Data for the *P. falciparum* and *P. yoelii* invasion were obtained during this study. Identical chamber slides were used for both *P. falciparum* and *P. yoelii*, and adjusted to the same RBC concentration (Table S1).
(TIF)

Table S2 **Morphological transition of invasive and non-invasive merozoites in two** ***Plasmodium*** **species after released from RBC.** The major axis, minor axis, longitudinal cross section area, and circularity were measured every 10 sec from RBC rupture to pre-invasion for invasive merozoites. The merozoites that did not invade within 20 min after RBC rupture were defined counted as non-invasive merozoites.
(TIF)

Movie S1 **From merozoite release to RBC invasion by** ***Plasmodium yoelii*** **17XL.**
(MP4)

Movie S2 **From merozoite release to RBC invasion by** ***Plasmodium yoelii*** **17X1.1.**
(MP4)

Movie S3 **From merozoite release to RBC invasion by** ***Plasmodium falciparum*** **3D7.**
(MP4)

Movie S4 **Lysed RBC membrane after invasion by** ***Plasmodium yoelii*** **17X1.1.**
(MP4)

## References

[1] Aikawa M, Miller LH, Johnson J, Rabbege J (1978) Erythrocyte entry by malarial parasites. A moving junction between erythrocyte and parasite. J Cell Biol 77, 72–82. 10.1083/jcb.77.1.72 PMC2110026 96121

[2] Cowman AF, Crabb BS (2006) Invasion of red blood cells by malaria parasites. Cell 124, 755–66. 10.1016/j.cell.2006.02.006 16497586

[3] Dvorak JA, Miller LH, Whitehouse WC, Shiroishi T (1975) Invasion of erythrocytes by malaria merozoites. Science 187, 748–750. 10.1126/science.803712 803712

[4] Gilson PR, Crabb BS (2009) Morphology and kinetics of the three distinct phases of red blood cell invasion by *Plasmodium falciparum* merozoites. Int J Parasitol 39, 91–6. 10.1016/j.ijpara.2008.09.007 18952091

[5] Baum J, Gilberger T, Frischknecht F, Meissner M (2008) Host-cell invasion by malaria parasites: insights from *Plasmodium* and *Toxoplasma*. Trends Parasitol 24, 557–63. 10.1016/j.pt.2008.08.006 18835222

[6] Treeck M, Zacherl S, Herrmann S, Cabrera A, Kono M, et al (2009) Functional analysis of the leading malaria vaccine candidate AMA-1 reveals an essential role for the cytoplasmic domain in the invasion process. PLoS Pathog 5: e1000322. 19283086 10.1371/journal.ppat.1000322 PMC2654807

[7] Leykauf K, Treeck M, Gilson PR, Nebl T, Braulke T, et al (2010) Protein kinase a dependent phosphorylation of apical membrane antigen 1 plays an important role in erythrocyte invasion by the malaria parasite. PLoS Pathog 6: e1000941. 20532217 10.1371/journal.ppat.1000941 PMC2880582

[8] van Dijk MR, Waters AP, Janse CJ (1995) Stable transfection of malaria parasite blood stages. Science 268, 1358–62. 10.1126/science.7761856 7761856

[9] Mota MM, Thathy V, Nussenzweig RS, Nussenzweig V (2001) Gene targeting in the rodent malaria parasite *Plasmodium yoelii*. Mol Biochem Parasitol 113, 271–8. 10.1016/s0166-6851(01)00228-6 11295181

[10] Yoeli M, Hargreaves BJ (1974) Brain capillary blockage produced by a virulent line of rodent malaria. Science 184, 572–3. 10.1126/science.184.4136.572 4595458

[11] Yoeli M, Hargreave B, Carter R, Walliker D (1975) Sudden increase in virulence in a strain of *Plasmodium berghei yoelii* . Ann Trop Med Parasitol 69(2): 173–8. 1098585 10.1080/00034983.1975.11686998

[12] Li J, Pattaradilokrat S, Zhu F, Jiang H, Liu S, et al. (2011) Linkage maps from multiple genetic crosses and loci linked to growth-related virulent phenotype in *Plasmodium yoelii*. Proc Natl Acad Sci USA 108, E374–82. 10.1073/pnas.1102261108 PMC3150948 21690382

[13] Carlton JM, Angiuoli SV, Suh BB, Kooij TW, Pertea M, et al. (2002) Genome sequence and comparative analysis of the model rodent malaria parasite *Plasmodium yoelii yoelii*. Nature 419, 512–9. 10.1038/nature01099 12368865

[14] Janse CJ, Ramesar J, Waters AP (2006) High-efficiency transfection and drug selection of genetically transformed blood stages of the rodent malaria parasite *Plasmodium berghei*. Nat Protoc 1, 346–56. 10.1038/nprot.2006.53 17406255

[15] Jongco AM, Ting LM, Thathy V, Mota MM, Kim K (2006) Improved transfection and new selectable markers for the rodent malaria parasite *Plasmodium yoelii*. Mol Biochem Parasitol 146, 242–50. 10.1016/j.molbiopara.2006.01.001 16458371

[16] Spence P J, Cunningham D, Jarra W, Lawton J, Langhorne J, et al. (2011) Transformation of the rodent malaria parasite *Plasmodium chabaudi*. Nat Protoc 4, 553–61. 10.1038/nprot.2011.313 PMC3968397 21455190

[17] Kendric RK, Peters W (1978) Rodent Malaria, Academic Press Inc. London LTD., 85–168.

[18] Liu J, Guo X, Mohandas N, Chasis JA, An X (2010) Membrane remodeling during reticulocyte maturation. Blood 115, 2021–7. 10.1182/blood-2009-08-241182 PMC2837329 20038785

[19] Glushakova S, Yin D, Li T, Zimmerberg J (2005) Membrane transformation during malaria parasite release from human red blood cells. Curr Biol 15, 1645–50. 10.1016/j.cub.2005.07.067 16169486

[20] Blackman MJ, Fujioka H, Stafford WH, Sajid M, Clough B, et al (1998) A subtilisin-like protein in secretory organelles of *Plasmodium falciparum* merozoites. J Biol Chem 273(36): 23398–409. 9722575 10.1074/jbc.273.36.23398

[21] Blackman MJ (2008) Malarial proteases and host cell rupture: an ‘emerging’ cascade. Cell Microbiol 10, 1925–34. 10.1111/j.1462-5822.2008.01176.x PMC2610400 18503638

[22] Singh S, Alam MM, Pal-Bhowmick I, Brzostowski JA, Chitnis CE (2010) Distinct external signals trigger sequential release of apical organelles during erythrocyte invasion by malaria parasites. PLoS Pathog 6: e1000746. 20140184 10.1371/journal.ppat.1000746 PMC2816683

[23] Peterson MG, Marshall VM, Smythe JA, Crewther PE, Lew A, et al. (1989) Integral membrane protein located in the apical complex of *Plasmodium falciparum*. Mol Cell Biol 9, 3151–4. 10.1128/mcb.9.7.3151-3154.1989 PMC362792 2701947

[24] Narum DL, Ogun SA, Thomas AW, Holder AA (2000) Immunization with parasite-derived apical membrane antigen 1 or passive immunization with a specific monoclonal antibody protects BALB/c mice against lethal *Plasmodium yoelii yoelii* YM blood-stage infection. Infect Immun 68, 2899–906. 10.1128/iai.68.5.2899-2906.2000 PMC97502 10768987

[25] Alexander DL, Arastu-Kapur S, Dubremetz JF, Boothroyd JC (2006) *Plasmodium falciparum* AMA1 binds a rhoptry neck protein homologous to TgRON4, a component of the moving junction in *Toxoplasma gondii*. Eukaryot Cell 5, 1169–1173. 10.1128/EC.00040-06 PMC1489286 16835460

[26] Collins CR, Withers-Martinez C, Hackett F, Blackman MJ (2009) An inhibitory antibody blocks interactions between components of the malarial invasion machinery. PLoS Pathog 5, e1000273. 10.1371/journal.ppat.1000273 PMC2621342 19165323

[27] Richard D, MacRaild CA, Riglar DT, Chan JA, Foley M, et al (2010) Interaction between *Plasmodium falciparum* apical membrane antigen 1 and the rhoptry neck protein complex defines a key step in the erythrocyte invasion process of malaria parasites. J Biol Chem 285(19): 14815–22. 20228060 10.1074/jbc.M109.080770 PMC2863225

[28] Bannister LH, Mitchell GH (1995) The role of the cytoskeleton in *Plasmodium falciparum* merozoite biology: an electron-microscopic view. Ann Trop Med Parasitol 89, 105–11. 10.1080/00034983.1995.11812940 7605118

[29] Kaneko O, Mu J, Tsuboi T, Su X, Torii M (2002) Gene structure and expression of a *Plasmodium falciparum* 220-kDa protein homologous to the *Plasmodium vivax* reticulocyte binding proteins. Mol Biochem Parasitol 121, 275–8. 10.1016/s0166-6851(02)00042-7 12034462

[30] Otsuki H, Kaneko O, Thongkukiatkul A, Tachibana M, Iriko H, et al. (2009) Single amino acid substitution in *Plasmodium yoelii* erythrocyte ligand determines its localization and controls parasite virulence. Proc Natl Acad Sci U S A 106, 7167–72. 10.1073/pnas.0811313106 PMC2678433 19346470

[31] Child MA, Epp C, Bujard H, Blackman MJ (2010) Regulated maturation of malaria merozoite surface protein-1 is essential for parasite growth. Mol Microbiol 78, 187–202. 10.1111/j.1365-2958.2010.07324.x PMC2995310 20735778

[32] Walliker D, Quakyi IA, Wellems TE, McCutchan TF, Szarfman A, et al (1987) Genetic analysis of the human malaria parasite Plasmodium falciparum. Science 1987 Jun 26 236(4809): 1661–6. 10.1126/science.3299700 3299700

[33] Trager W, Jensen JB (1976) Human malaria parasites in continuous culture. Science 193, 673–5. 10.1126/science.781840 781840

[34] Lambros C, Vanderberg JP (1979) Synchronization of *Plasmodium falciparum* erythrocytic stages in culture. J Parasitol 65, 418–20. 383936

